# Study design to evaluate cognitive behavioral therapy among a diverse sample of adults with a first-time DUI offense

**DOI:** 10.1186/s13722-016-0053-x

**Published:** 2016-03-31

**Authors:** Karen Chan Osilla, Katherine E. Watkins, Magdalena Kulesza, Karen Flórez, Marielena Lara-Greenberg, Jeremy N. V. Miles

**Affiliations:** RAND Corporation, 1776 Main Street, P.O. Box 2138, Santa Monica, CA 90407-2138 USA

**Keywords:** DUI, Cognitive behavioral therapy, Alcohol use disorders

## Abstract

**Background:**

Driving under the influence (DUI) of alcohol is a major public health concern, and many individuals continue to drink and drive even after being convicted of a DUI offense. Latinos, in particular, are disproportionately likely to be arrested for a DUI, have higher rates of recidivism, and are more likely to die in alcohol-related accidents than non-Latino Whites. Latinos also experience significant disparities in accessing alcohol-related treatment.

**Methods/design:**

This study protocol paper describes a randomized trial of cognitive behavioral therapy (CBT) compared to usual care in DUI programs for individuals with a first-time offense and at-risk drinking. We will utilize a two-group randomized design where individuals enrolled in a DUI program with a first-time conviction will be randomized to CBT (n = 150) or usual care (n = 150). Participants will be assessed at baseline, immediately post-treatment, and 6-months post-treatment. Recidivism data will be collected using administrative data within 2 years post-treatment.

**Discussion:**

This project has the potential to benefit a large population of vulnerable individuals who are at risk of DUI recidivism. It also develops a new model of care by providing treatment in DUI programs to reduce disparities associated with poor treatment access.

*Trial registration* NCT02588703

## Background

Preventing recidivism among individuals convicted of driving under the influence (DUI) is an important public health objective. Individuals convicted of a DUI often continue to drive while intoxicated [1–4]. Drivers with repeated offenses are also more likely to be involved in fatal crashes. In 2010, intoxicated drivers involved in fatal crashes were four times more likely to have a prior DUI conviction than drivers in fatal crashes who were not intoxicated [5].

Addressing DUI recidivism in the Latino population is particularly important because compared to other ethnic groups, Latinos are disproportionately more likely to be arrested for a DUI, have higher rates of recidivism, and are more likely to die in alcohol-related crashes [6–10]. Although some have noted important variations in these outcomes across socioeconomic status and place of birth, the patterns of consumption among certain Latino subgroups continues to be a cause for concern [11]. For example, in a sample from Texas, Latinos were more likely than whites to report more days of heavy drinking [9, 12]. Latinos are also significantly less likely than Whites to seek specialty alcohol or drug treatment programs, and these odds worsen with alcohol use severity [13–15]. Latinos face significant structural barriers to treatment access including stigma, financial worries, difficulty finding services, and having limited or no insurance [16, 17]. Innovative treatment models that address these barriers are needed to reduce this health disparity and address rates of alcohol use disorders (AUD) in this population [18].

One reason for the high rates of recidivism among both Latinos and other populations may be because many individuals convicted of a DUI have an unrecognized and untreated AUD [19], which typical interventions provided by DUI programs may not fully address. The majority of DUI program content consists of alcohol education or information about the consequences of drinking and driving [20]. Group counselling is also provided, though the content is not standardized across the program. Rates of AUD appear to increase with multiple DUI convictions [21] and in one study, continued to be present even 15 years after a first DUI [22]. Those with multiple DUI convictions are five times more likely to have a diagnosis of AUDs than the general population [22]. While these rates are high [23], rates of AUDs are likely higher than documented because of underreporting among those with repeat offenses [24]. There is a need to examine whether providing treatment for AUD to individuals attending DUI programs could decrease recidivism.

Numerous randomized controlled trials have demonstrated that CBT is an effective treatment for alcohol abuse and dependence [25–30], yet few studies have evaluated CBT in DUI programs [31–33]. Because Latinos are less likely than Whites to access specialty alcohol use treatment, providing cognitive behavioral therapy (CBT) within a mandatory DUI program may reduce disparities in treatment access and improve alcohol-related health outcomes. It may also improve physical and mental health-related functioning [31, 34–37] and decrease future injuries and alcohol-related hospitalizations [38–40]. CBT is a counselling approach that utilizes coping skills, problem solving, and cognitive restructuring to address how individuals’ thoughts, actions/behaviors, and feelings influence each other [41]. Combined with coping skills training, CBT is one of several highly ranked evidence-based treatment programs [42, 43]. Unfortunately, while several studies suggest that CBT is acceptable and effective among Latinos, these studies have focused on CBT for depression and not drinking [44–49].

To address the dual problem of DUI recidivism and the lack of alcohol treatment utilization by Latino populations, we designed a study to evaluate the efficacy of CBT in DUI programs. We describe the study protocol of a two-group randomized trial where individuals enrolled in a DUI program with a first-time conviction will be randomized to CBT or Usual Care (UC).

### Specific aims and hypotheses

The specific aims of this project are to: (1) evaluate the efficacy of CBT on reported heavy drinking, percent days abstinent, alcohol-related self-efficacy, and intent to drink and drive; and, (2) evaluate factors associated with improved treatment outcomes and 2-year DUI recidivism by examining whether race/ethnicity, gender, acculturation, and alcohol situational norms predict treatment outcomes and DUI recidivism. We hypothesize that those randomized to CBT will have greater improvements in alcohol-related outcomes at 6-month follow-up and lower likelihood of recidivism two years later than those in UC.

## Methods/design

### Overview of study procedures

All procedures have been approved by the Institution’s Review Board. There are two phases to the study. The first phase will be focused on adapting the CBT treatment for the DUI population by eliciting feedback about each session from focus group participants. The second phase will be a randomized trial of the adapted 9-session CBT treatment compared to Usual Care. Potential participants will be screened for at-risk drinking using the AUDIT-C [four or more (if male) or three or more (if female or other)] [50], randomized to either UC or CBT, and then asked to complete self-administered assessments at baseline, immediately post-treatment (i.e., 3-months post-baseline), and 6-months post-treatment. We will also assess recidivism up to 48 months after the treatment ends using administrative data collected by the State of California. Analyses will be conducted to compare overall group efficacy between CBT and UC. We will also analyze predictors of treatment response and recidivism by examining whether race/ethnicity, gender, acculturation, and alcohol situational norms predict treatment outcomes and DUI recidivism.

### Study setting

We will draw upon an existing collaboration with the Los Angeles County Substance Abuse Prevention and Control (LAC-SAPC) and three private DUI programs under LAC-SAPC’s regulatory authority. In Los Angeles County, when an adult is arrested for DUI for the first time, in addition to having a suspended driver’s license and other sanctions, the individual must also attend a 3-month DUI program that meets California’s Title 9 requirements [51].

The three participating DUI programs provide English and Spanish-speaking services to diverse clientele. Individuals enrolled in a 3-month DUI program attend a minimum of 12 h of alcohol and drug-related educational classes, 18 h of group counselling, and three individual assessment interviews [51]. Educational classes focus on the effects of alcohol and other drugs; nature of addiction to alcohol and drugs; impairment of driving abilities, skills, and judgment caused by consumption of alcohol or drugs; alternatives to the abuse of alcohol and drugs; and the effects of alcohol or drug use on the individual, family, and society. Group counselling focuses on encouraging clients to share ideas, examine their personal attitudes and behavior, and provide support for any positive changes. Of note, no specific guidance on the content or style of the counselling groups is given by State law so it is unknown whether these groups provide evidence-based treatment. Face-to-face individual interviews occur at the beginning, midpoint, and end of the program to monitor fee payment, attendance, barriers to program completion, and assessment of additional services needed [52].

### Participants

Only English-speaking clients will be recruited based on our previous work showing small proportions of Spanish-only speaking clients in our participating programs [19, 53]. Participants will be English-speaking individuals 21 and older who are convicted of a first-time DUI offense and enter one of the three participating 3-month DUI programs in LA County. We will recruit individuals with first-time offenses rather than individuals with a repeat offense to control for treatment dose, as individuals with repeat offenses attend longer programs. Participants will be screened for at-risk drinking using the AUDIT-C [50].

We plan to recruit all eligible individuals with at-risk drinking who have been convicted of a first-time DUI regardless of ethnicity because we do not want to exclude clients from the possibility of treatment while in the DUI program. We expect that our population for sampling will be 65 % male; 50 % Hispanic/Latino, and 10 % African American.

### Description of CBT treatment

The proposed CBT treatment [32] has been shown to be effective in reducing drinking and improving coping skills across several randomized trials [32, 54–57], and has been adapted as an individualized treatment for AUDs in Project MATCH [58]. The treatment is designed to be delivered in a group format for nine 90-min sessions with two optional sessions on nonverbal communication and assertiveness [32].

To meet Title 9 requirements and to keep treatment dosage the same as UC, the current study will deliver nine sessions once per week, each lasting 2-h (see Table 1). The goals of the CBT treatment will be to reduce drinking and related consequences including drinking and driving.

**Table 1:** Proposed CBT sessions

Session	Session topic
1	Making informed choices about drinking
2	Managing urges to drink
3	Connecting situations, thoughts, and feelings
4	Managing negative thinking
5	Receiving criticism about drinking
6	Seemingly irrelevant decisions
7	Communication skills
8	Developing social support networks
9	Increasing pleasant activities

- *Session 1* Counsellors will focus on educational information about alcohol and alcohol’s effects on decision making in order to assist participants in their ability to make informed decisions and how much to drink [59].
- *Session 2* Participants will learn to identify and cope with different high-risk situations that can increase the likelihood of drinking and driving [32, 60].
- *Session 3* Counsellors will help participants understand the connection between thoughts, feelings, and behavior as it relates to drinking in particular [60]. Participants will be introduced to how their thoughts and feelings are directly connected with their behaviors.
- *Session 4* Participants will learn helpful strategies to identify and manage their negative thinking using cognitive restructuring techniques. Participants will complete a cognitive restructuring chart called “Catch it, Check it, Change it” [60] that helps participants identify and change harmful thoughts that lead to drinking and driving.
- *Session 5* Participants will be introduced to skills that will help them cope with criticism from others about their drinking [32]. Participants will learn different types of criticism and strategies to cope with each (e.g., react less defensively, find something to agree with, work out a compromise).
- *Session 6* Participants will learn how to identify seemingly irrelevant decisions that lead to drinking and/or drinking and driving [32]. Participants will learn that these decisions are ordinary choices we make every day that eventually lead to drinking and/or drinking and driving. Participants will complete a chaining exercise examining the decisions that lead up to their last drinking and/or drinking and driving episode.
- *Session 7* Participants will learn useful skills that will help them improve their communication, and thus increase their friendships and supportive network. They will learn the differences between passive, aggressive, passive-aggressive, as well as assertive communication, and will role-play asking assertive requests.
- *Sessions 8 and 9* Counsellors will discuss helpful strategies to increase their support network and enjoyable non-drinking activities that can be accomplished either alone or with others.

In each session, the rationale for the session will first be described as it relates to drinking behavior and preventing future DUI, and then the content of the module or “skill guidelines” will be reviewed. The counsellor and participants then practice skills using role plays and group exercises. Because each session is self-contained and includes a review of the rationale and skill guidelines, the group can have “rolling admission,” in which members can join at any session in the treatment. Rolling CBT groups are common and more sustainable in community settings [44–49], which makes the proposed CBT groups more comparable to UC.

### Phase 1 Procedures

The purpose of Phase 1 is to adapt the CBT manual to be suitable for CBT group treatment in DUI programs and receive feedback from DUI program clients to determine if it is appropriate and helpful.

#### CBT conceptual framework

CBT extends cognitive therapy through use of behavioral techniques [61]. Cognitive therapy is based on a model that suggests that “distorted or dysfunctional thinking (which influences the patient’s mood and behavior) is common to all psychological disturbance” [62]. This cognitive model is common across treatments for depression and substance use. The model posits that substance abuse is perpetuated by maladaptive thoughts and beliefs [63]. Core techniques of CBT, such as emotional regulation and positive reframing, have been adapted to be culturally meaningful for Latinos (e.g., use of imagery involving light, which is analogous to Christian imagery embraced by some Latinos; reference to social/peer/family pressure, familism, and fatalism) and have been found to improve retention and treatment outcomes in Latino populations [46, 64, 65].

The central tenets of the CBT treatment are based on improving coping skills [32]. Lack of coping skills can increase the likelihood of drinking when confronted with high-risk scenarios such as feeling stressed or attending a social gathering where alcohol is served [66–68]; thus, the primary goal of the CBT treatment is to reduce negative consequences that arise from drinking behavior and drinking and driving by increasing awareness of potential high-risk situations through the use of coping skills.

#### CBT group treatment adaptation

In Phase 1, we will follow adaptation procedures from our previous research [53, 69, 70] to ensure that the CBT treatment is relevant for diverse DUI clients and feasible to administer within the group DUI setting. We will follow Barrera and Castro’s [71] framework, which includes information gathering from key informants (i.e., DUI clients, counsellors, administrators), preliminary adaptation of the CBT manual, pilot testing using focus groups with DUI clients, and further revisions incorporating client feedback from the focus groups. Our goal is to ensure that the CBT treatment is compatible for clients who have different cultural patterns, meanings, and values [72], and that we maintain fidelity to the core elements of CBT. We will gather information by conducting approximately ten focus groups with DUI clients. These focus groups will consist of 8–10 existing clients recruited from DUI usual care groups currently in session. We will first conduct one to two focus groups prior to adapting the CBT manual to obtain general reactions about the CBT model, potential session topics, and important cultural or other adaptations we need to account for when adapting the CBT treatment (e.g., language describing drinking and drinking and driving). During the focus groups, we will demonstrate parts of the CBT sessions and then ask clients for their feedback. Their feedback will be elicited in two main sections: (1) Acceptability and feasibility of the CBT group material (e.g., “Which messages were the most convincing to reduce drinking while driving? Least convincing? What was the most helpful information that you saw? Least helpful? What do you think would help other clients?”); and, (2) Cultural acceptability of the treatment content (e.g., “How can we make the program helpful to people from different backgrounds? What messages, images, or phrases are most meaningful, within and across cultures?”). We will iteratively make changes to our treatment manual following each focus group. Our final step in the treatment adaptation process will be to revise our manual to incorporate suggestions commonly brought up across the focus groups.

#### Focus group recruitment

Recruitment for the focus groups will use effective strategies from our previous research [19, 53], and the targeted recruitment of English speakers is consistent with the population that attends LA County DUI programs. We will be recruiting clients already enrolled in DUI Usual Care because we want feedback about how their experiences in Usual Care compares with the CBT group information we are presenting; we also want opinions from a variety of clients at different phases in the program (e.g., just started program, about to end program) who can provide insight on what information is helpful or unhelpful in reducing risk of DUI recidivism. DUI counsellors will briefly describe the study and pass out consent-to-contact forms. Clients will be asked to return their form in an envelope (whether blank or completed) to avoid feelings of coercion to participate. Envelopes will be given to their counsellor who will return them to research staff. Research staff will call clients who consent to be contacted, to schedule an in-person appointment to obtain informed consent for study participation.

### Phase 1 analysis plan

Focus groups will be audio recorded. Collection and interpretation of focus group data will follow approaches we have used in previous work [53, 73, 74]. Following grounded theory analyses [75], we will discuss each category and generate underlying themes. Classic content analysis will be used to identify quotes that fit each theme [76, 77]. Then, we will sort quotes by theme and reach a consensus on any discrepancies. This analysis will allow us to understand feasibility and acceptability, and will inform the delivery of CBT in a diverse DUI setting.

### Phase 2 procedures

The purpose of Phase 2 is to determine the efficacy of a 9-session CBT group treatment for AUD compared to UC among individuals who are court-ordered to attend a 3-month DUI program for a first-time conviction. We will also examine predictors of CBT efficacy to further understand the characteristics of participants who benefit most from treatment.

**Fig. 1:**
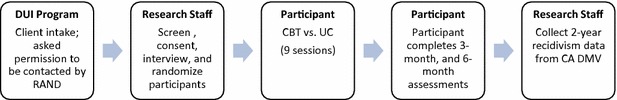
Intervention and data collection flow

Participants (N = 300) will be recruited at intake (see Fig. 1). Upon enrolling in the DUI program, program staff will ask clients if research staff can contact them about a research study. Interested clients will complete a consent-to-contact form. After DUI program staff has completed their procedures, on-site research staff will screen, obtain consents, and randomize potential clients. For rare instances when staff is unavailable, DUI staff will fax consent-to-contact forms to a secure fax machine and research staff will screen and gain consent from the client by phone. Staff will screen for at-risk drinking using the AUDIT-C [four or more (if male) or three or more (if female or other)] [50]. Participants will be asked to complete a baseline survey, post-treatment survey at DUI program completion (i.e., 3-months post-baseline), and a follow-up survey 6 months after program completion. Participants will receive a $25 gift card for the baseline survey, $25 for the post-survey, and $50 for the 6-month follow-up. All participants will be followed, regardless of whether they complete UC or CBT, and we will conduct intent to treat analyses.

#### Flow rates

Based on our previous research recruiting DUI clients [19], we expect 28 new DUI program clients to enroll at the collaborating DUI programs each week. We estimate that 72 % of clients will agree to be contacted by RAND, 70 % will be eligible for the study after screening, 81 % will agree to participate in the study, 82 % will drop out of the DUI program, and 85 % will be retained at the 6-month follow-up [19].

We will examine recidivism data obtained from the California’s Department of Motor Vehicles (DMV) indicating whether, within the two years after treatment, participants in our study recidivated, which we define as having a subsequent alcohol-related violation (i.e., DUI convictions, alcohol-related incidents and license actions, traffic violations including moving violations). We will also examine secondary measures of recidivism including alcohol-related injuries and crashes by obtaining the number of alcohol-related injuries and crashes/fatalities within the assessment period.

We will implement randomized block assignment within equally-sized strata defined by DUI program to assign 300 individuals to one of two conditions: CBT or UC. Within each DUI program, randomization will occur within random blocks of size 6, thereby ensuring the number of people allocated to each group is approximately equal throughout recruitment and making it very difficult to determine the randomization of the next participant [78]. The randomization sequence will be developed in advance using the blockrand package [79]. This will result in 150 clients within each condition. Participants will enter groups on a rolling basis, which means that participants can enter the next available group (per DUI program procedures).

#### CBT group counsellors and training

Counsellors in the DUI program are registered through state certification and have at least two years of experience in substance abuse counselling. We will use a training model that was used successfully in our previous implementation of CBT treatment [80]. DUI counsellors will be trained to deliver the CBT through a 2-day didactic training, followed by supervised practice in conducting the entire 9-session treatment (the training phase) with DUI program clients in the three DUI programs, followed by a 1-day didactic refresher training. Training will include three parts: (1) An introduction to the CBT model including theory of how thoughts, behaviors, and alcohol use interact; (2) A detailed review of each of the nine weekly sessions in the treatment manual followed by a counsellor role-play session, including feedback from trainers and group participants; and (3) A discussion of sensitive issues that may arise in the group (e.g., group management issues, such as how to balance the talking among participants, how to handle participants at different stages of change with their drinking, clinical issues such as intimate partner violence, and emergencies such as suicide ideation). Counsellors will participate in weekly, individual or group supervision meetings. Audio recordings will be reviewed during supervision meetings to support ongoing adherence and to highlight areas for improvement.

#### CBT group fidelity and adherence monitoring

We will use methods from our previous studies to assess the fidelity of CBT and adherence to session content [81]. We will randomly select 15 % of CBT session recordings to be coded by trained CBT coders. Two individuals with Masters Degrees in either psychology or public health/nursing will be recruited to code. They will receive 24 h of training (covering the nine CBT sessions). Coder training will review treatment materials, key points of the sessions, and guidelines of the rating form, which will be adapted from the Yale Adherence and Competence Scale (YACS; [82]). The YACS [82–85] has been utilized in large clinical trials such as Project MATCH [83, 84]. Coders rate therapist adherence and competence in delivering manualized treatments for alcohol and other drug use related problems [82, 86]. We will estimate interrater reliability using the intraclass correlation coefficient (ICC [87]). The fidelity measure will allow us to describe counsellors’ fidelity to CBT and adherence to the treatment, and whether the counsellors are competent in delivering the CBT. We anticipate that counsellors will demonstrate high fidelity prior to the start of the efficacy trial, and that there will be little variability during implementation due to ongoing supervision [81]. Therefore, we do not evaluate the relationship between fidelity and outcomes (Table 2).

**Table 2:** Data collection measures

Measure	Instrument	Baseline	3 months	6 months
Alcohol use: days abstinent, heavy drinking; typical quantity, frequency^a^	DDQ	X	X	X
Alcohol-related self-efficacy^b^	AASE	X	X	X
Intent to drink and drive^b^	BADDS	X	X	X
Alcohol-related negative consequences^b^	SIP-AD	X	X	X
Alcohol-related health consequences (i.e., injuries, hospitalizations)^b^	NSDUH	X	X	X
Attitudes about drinking and driving^b^	BADDS	X	X	X
Demographic information^c^	Sociodemographic form	X		
Acculturation^c^	PAS-3	X		
Alcohol situational norms^c^	ASN	X	X	X

*DDQ* Daily Drinking Questionnaire, *AASE* Alcohol Abstinence Self-Efficacy Scale, *BADDS* Behaviors and Attitudes Drinking and Driving Scale, *NSDUH* National Survey on Drug Use and Health (NSDUH), *SIP*-*AD* Short Inventory of Problems Alcohol and Drugs (adapted for alcohol only in this study), *PAS*-*3* Proxy Acculturation Scale, *ASN* alcohol situational norms

^a^Primary outcome

^b^Secondary outcome

^c^Covariate

### Phase 2 analysis plan

We will use an intent-to-treat (ITT) approach, in which all persons who complete baseline will be assessed. Thus, the overall efficacy of the treatment will include the effect of attrition and non-compliance. DUI programs will not use special procedures to keep clients in our experimental treatment. Analyses will use the standard ITT approach to examine the effect of offering the CBT to all participants. Our ITT approach will analyze participants as belonging to the group they were randomized to, regardless of their compliance or attrition, because excluding clients that do not attend the CBT sessions would be likely to bias our results in favor of the CBT [88].

Our primary analysis will be to examine differences in the primary outcome (rates of heavy drinking and percent days abstinent) at both follow-ups controlling for baseline and other characteristics (age, gender, ethnicity; see Table 2). Similar analyses will be conducted for the secondary (self-efficacy, intent to drink and drive) outcomes. The rolling group design violates the independence assumption [89, 90]. Analyses will adjust for the rolling group design using a multiple membership model [91].

### Phase 3 Procedures

In Phase 3, we will explore whether gender, race/ethnicity, acculturation, and alcohol situational norms are predictors of our primary treatment outcomes (rates of heavy drinking, and percent days abstinent) and DUI recidivism (alcohol-related violations). Such analyses will allow us to leverage the dataset to explore predictors of behavior change, thereby improving the development of future treatments. Examining differences by race/ethnicity and other sociocultural characteristics will allow us to examine whether racial/ethnic disparities are reduced, which is a research priority of the Institute of Medicine [92].

### Phase 3 analysis plan

We will use multilevel logistic regression to analyze the relationship between the measured predictors (gender, age, acculturation, alcohol situational norms) and recidivism (alcohol-related violations). Based on 2013’s DUI recidivism data in California [2], about 6 % of individuals with a DUI arrest experienced a subsequent crash or violation that was alcohol-related within 12 months. Thus, we acknowledge that we may not have the power to detect differences without a large sample size (e.g., 1500/group). We will instead examine whether predictors of heavy drinking (gender, race/ethnicity, age, acculturation, alcohol situational norms) are predictive of recidivism (i.e., alcohol-related violations). We choose predictors of heavy drinking because there is a lack of research examining predictors of recidivism among Latinos. These findings will assist with the development and refinement of future treatments in this population. The rare nature of alcohol-related violations and crashes [2] means that sparse data may cause estimation problems due to separation issues and our models may be limited. Given the limitations on the models that can be analyzed, we recognize this aim is exploratory and that a power analysis is at risk of providing excess confidence in our analysis.

## Discussion

The proposed study addresses at least two important public health problems: (1) Despite the fact that individuals convicted of a DUI are mandated to attend a DUI program, many recidivate and, (2) There is a disproportionate lack of access to AUD treatment for Latino populations. Our study addresses these problems in the following ways. First, by providing an evidence-based treatment for AUDs in a DUI setting, we aim to decrease recidivism, as evidence suggests that untreated AUDs are an important risk factor for recidivism. Providing treatment in this setting may be especially important for people at high risk of recidivism, given that they are more likely to underreport and minimize risky alcohol use [24] and may not attend referrals to more intensive specialty care [23]. Second, we hope to address disparities in AUD treatment access experienced by Latinos, as Latinos are overrepresented in DUI programs and underrepresented in treatment programs. By testing the efficacy of treatment that is integrated within the DUI program, our study will increase the reach of healthcare services for individuals with AUDs who may have otherwise gone without treatment.

We anticipate several challenges to the implementation of the proposed treatment protocol. First, we anticipate that it will take an extended amount of time to train existing counsellors to proficiency. Counsellors will have existing experience that may conflict with the CBT treatment philosophy and the challenge of delivering a structured treatment may be difficult for some to follow. Second, the flow of participant recruitment is always difficult to predict despite previous studies and experiences establishing workflow. We therefore build in cushion into the timeline to effectively address potential problems with these challenges including the possibility of hiring different counsellors and/or engaging additional DUI programs to meet the study’s needs.

This study is limited to an English-speaking sample of first-time offenders. Future studies may consider evaluating CBT in other DUI programs in other populations that have higher concentrations of Spanish monolingual speakers and greater variability in Latino subgroups to evaluate whether or not CBT is helpful. In addition, studies could examine the effectiveness of CBT with repeat offenders. The current study also engages only those offenders that attend DUI programs and future studies may consider earlier preventive interventions to reach those who engage in drinking and driving, but do not access DUI programs (e.g., those who are convicted, but choose not to reinstate their license; those who are arrested but not convicted). Finally, we acknowledge that this study does not address contextual (profiling) and structural (insurance) factors that influence drinking and driving behavior that would be significant contributions to the literature. Through extending the current study to address these noted limitations, future work using this CBT treatment is positioned to add a valuable contribution to both extant literature and well-being of many diverse communities affected by DUI and alcohol-related problems. Untreated AUD can have terrible consequences for the individual, their families, and society. Our work develops a novel approach to address DUI recidivism among a diverse population who may not otherwise access treatment.
